# The reproducibility debate is an opportunity, not a crisis

**DOI:** 10.1186/s13104-022-05942-3

**Published:** 2022-02-10

**Authors:** Marcus R. Munafò, Chris Chambers, Alexandra Collins, Laura Fortunato, Malcolm Macleod

**Affiliations:** 1 School of Psychological Science, University of Bristol, 12a Priory Road, Bristol, BS8 1TU UK; 2 MRC Integrative Epidemiology Unit at the University of Bristol, Bristol, UK; 3 Cardiff University Brain Research Imaging Centre (CUBRIC), School of Psychology, Cardiff University, Cardiff, UK; 4 Centre for Environmental Policy, Imperial College London, London, UK; 5 Institute of Human Sciences, University of Oxford, Oxford, UK; 6 Santa Fe Institute, Santa Fe, NM USA; 7 Centre for Clinical Brain Sciences, University of Edinburgh, Edinburgh, UK

**Keywords:** UK Reproducibility Network, Reproducibility, Replicability, Research Integrity

## Abstract

There are many factors that contribute to the reproducibility and replicability of scientific research. There is a need to understand the research ecosystem, and improvements will require combined efforts across all parts of this ecosystem. National structures can play an important role in coordinating these efforts, working collaboratively with researchers, institutions, funders, publishers, learned societies and other sectoral organisations, and providing a monitoring and reporting function. Whilst many new ways of working and emerging innovations hold a great deal of promise, it will be important to invest in meta-research activity to ensure that these approaches are evidence based, work as intended, and do not have unintended consequences. Addressing reproducibility will require working collaboratively across the research ecosystem to share best practice and to make the most effective use of resources. The UK Reproducibility Network (UKRN) brings together Local Networks of researchers, Institutions, and External Stakeholders (funders, publishers, learned societies and other sectoral organisations), to coordinate action on reproducibility and work to ensure the UK retains its place as a centre for world-leading research. This activity is coordinated by the UKRN Steering Group. We consider this structure as valuable, bringing together a range of voices at a range of levels to support the combined efforts required to enact change.

## Introduction

The UK Reproducibility Network (UKRN) is a peer-led consortium that aims to ensure the UK retains its place as a centre for world-leading research [1]. The distinct elements of UKRN (described below) each prepared submissions to the UK Parliament Science and Technology Committee inquiry on reproducibility and research integrity [2], where they address some of the questions posed by this inquiry—in particular, the role of funders, institutions, researchers, publishers, and other organisations, and the potential impact of a UK Committee on Research Integrity. The UKRN Steering Group synthesised common themes across these in their submission, and we present our interpretation of these here.

In our view, the “reproducibility crisis” narrative can be unhelpful, in part because it implies an acute situation that can be resolved, whereas a framework for continuous improvement will be necessary and ultimately more effective to ensure a healthy research culture. Whilst there are many reasons why specific research findings may not be robust, we agree that there is considerable room for improving the quality of research outputs, and that this should generate improvements in the speed with which knowledge is generated, and in turn provide benefit to society.

The UKRN comprises three main elements: (1) Local Networks, which comprise informal, self-organising groups of researchers interested in isses of reproducibility, replicability and research quality, (2) Institutional Members, who have created senior academic positions focused on research improvement, and (3) External Stakeholder, which comprises funders, publishers, learned societies and other sectoral organisations. The activity across and between these elements, as well as the overall strategic direction of UKRN, is coordinated by a Steering Group.

The UKRN structure reflects the inter-connected nature of the research ecosystem. It is also intended to ensure that initiatives aimed at improving research quality are *informed* by the voice of the grassroots research community and the needs of other stakeholders (e.g., funders), and that they are *coordinated*. It also represents an attempt to work collectively and collaboratively (rather than competitively). In our view, whilst a degree of competition is healthy, the extent to which academia has become hyper-competitive is problematic at both an individual and institutional level, and contributes to problems of low reproducibility and replicability.

## Main text

As we have written previously: “Modern research-intensive universities present a paradox. On the one hand, they are dynamic, vibrant institutions where researchers use cutting-edge methods to advance knowledge. On the other, their traditions, structures, and ways of working remain rooted in the nineteenth century model of the independent scientist” [3]. In our view this underlying culture, where research groups operate effectively as artisanal small businesses, when combined with human cognitive biases and the current incentive structures, interact to contribute to problems of poor reproducibility and replicability.

The research ecosystem is therefore complex and highly interconnected. Incentives, for example, may be embodied in institutional hiring and promotion practices, but are also influenced by the demands of research funders, the (explicit or implicit) requirements of journals and publishers, and the enthusiasm of researchers themselves to make discoveries that will advance their field. In the majority of cases, these influences are unconscious or implicit, and while introduced for the best of reasons, they often have unintended consequences that have a negative impact on research quality.

To bring about change, efforts will need to be coordinated across the research ecosystem [4]. For example, open research practices (e.g., sharing data and code) will require supporting digital infrastructures, training in the skills necessary to make use of these infrastructures, mandates from funders and publishers to require open research practices where appropriate, monitoring of performance to create motivation for improvement (which in turn requires coordination across institutions, funders and publishers), and recognition of these practices in institutional hiring and promotion processes (again to create an incentive).

In other words, no one actor in the research ecosystem can bring about meaningful change on their own. Indeed, individual actors may place themselves at a disadvantage if they work in ways that benefit their research, if this is not aligned with what is rewarded within current incentive structures. For example, whilst there are advantages to individual researchers in engaging in open research practices [5], institutional recognition of this will be necessary for career advancement. And if this recognition is not sector-wide, then a researcher working in alignment with their own institutional framework may be at a relative disadvantage when moving to another institution if such practices are not valued there.

There is therefore a need—for example, through meta-research activity—to understand how the research *system* affects the quality and robustness of research outputs, and consider how a programme of measures across different sectors can work in concert to improve research quality. We need to distinguish between *research* integrity (the processes, working practices and incentives that contribute to the quality of research outputs) and *researcher* integrity (i.e., the specific behaviour of individual researchers). Whilst both are important, we feel that focusing on the former (i.e., the system) will generate more benefits.

## The need for coordination

In our view, sectoral efforts to improve research quality across the research ecosystem require a focus on the research environment and the process of research. For example, open research practices are a possible means by which the quality of research can be improved. These practices also allow for more granular contributions to the research process (i.e., the component parts of a research workflow, such as the study protocol, data, analysis code, etc.) to be recognised. However, embedding and incentivising these practices will require coordination with researchers, institutions, funders, publishers, learned societies and a range of other sectoral organisations (including industry).

Critically, these efforts will require investment (e.g., in digital infrastructures and training to support open research practices), along with a more collaborative approach. Currently, local solutions (e.g., training, approaches to research assessment) are often developed by individual institutions and organisations. This is inefficient and reduces interoperability if it leads to variation across institutions, when a common approach that draws on a common model may be more efficient and effective. To this end, UKRN is producing statements for institutional use, for rapid adaptation and adoption (https://www.ukrn.org/common-statements/). We are also developing train-the-trainer courses on open research practices that allow coordinated training to be delivered locally in a way that ensures a degree of common approach. Funding initiatives that enable the sharing of resources and best practices across disciplines and institutions will allow continued and widening support for collaborative approaches.

In addition, activity of this kind should be evaluated and evidenced. There is an exciting level of innovation in the research ecosystem at present, including new publishing models (e.g., https://octopuspublishing.org/) and partnerships between funders and journals [6, 7]. However, innovations and approaches that *prima facie* appear likely to produce positive change may not deliver this in practice, or may have unintended consequences. Funding to support meta-research activity to evaluate the impact of new ways of working, and ensure that any benefits are understood and evidenced (and any potential unintended consequences monitored), will therefore be necessary. Such research should exploit the full range of tools available, including randomised controlled trials of effectiveness, and could be coordinated across research funders to ensure efficient use of resources.

This again speaks to the need for coordination, to identify where there is a need for such activity, and to ensure this activity is well targeted. National structures can play a critical role in these efforts, working collaboratively with researchers, institutions, funders, publishers, learned societies and other sectoral organisations. UKRN attempts to achieve this, but structures established by governments and funders, such as the planned UK Committee on Research Integrity, will bring additional weight to these efforts, and be able to provide other functions (e.g., monitoring and reporting).

## Outlook

Rather than viewing the current debate around the reproducibility and replicability of research findings as a “crisis”, it is more constructive in our view to frame it as an opportunity to reflect on which aspects of relevant working practices continue to be effective, which can be improved, and which new ways of working can beneficially be introduced to the research ecosystem. A systems view of the factors affecting research quality is required, with a coordinated approach across disciplines, institutions and sectors. Doing this effectively will require investment and ongoing evaluation—with the support of governments and funders—allowing us to move to a model of continuous improvement at the level of the individual researcher, the institution, sectoral organisations, and the sector as a whole.

## Data Availability

Not applicable.

## Abbreviation

UKRN: UK Reproducibility Network
